# Polarization-sensitive optical coherence tomography monitoring of percutaneous radiofrequency ablation in left atrium of living swine

**DOI:** 10.1038/s41598-021-03724-8

**Published:** 2021-12-21

**Authors:** Xiaowei Zhao, Ohad Ziv, Reza Mohammadpour, Benjamin Crosby, Walter J. Hoyt, Michael W. Jenkins, Christopher Snyder, Christine Hendon, Kenneth R. Laurita, Andrew M. Rollins

**Affiliations:** 1 Department of Biomedical Engineering, Case Western Reserve University, Cleveland, OH USA; 2 School of Medicine, Case Western Reserve University, Cleveland, OH USA; 3 Heart and Vascular Research Center, MetroHealth Medical Center, Cleveland, OH USA; 4 Department of Chemistry, Case Western Reserve University, Cleveland, OH USA; 5 The Congenital Heart Collaborative, Rainbow Babies and Children’s Hospital, Cleveland, OH USA; 6 Department of Pediatrics, Case Western Reserve University, Cleveland, OH USA; 7 Case Western Reserve University, Cleveland, OH USA; 8 Department of Electrical Engineering, Columbia University, New York, NY USA; 9 Department of Pediatrics, Ochsner Health, New Orleans, LA USA

**Keywords:** Cardiac device therapy, Imaging and sensing, Atrial fibrillation

## Abstract

Radiofrequency ablation (RFA) is commonly used to treat atrial fibrillation (AF). However, the outcome is often compromised due to the lack of direct real-time feedback to assess lesion transmurality. In this work, we evaluated the ability of polarization-sensitive optical coherence tomography (PSOCT) to measure cardiac wall thickness and assess RF lesion transmurality during left atrium (LA) RFA procedures. Quantitative transmural lesion criteria using PSOCT images were determined ex vivo using an integrated PSOCT-RFA catheter and fresh swine hearts. LA wall thickness of living swine was measured with PSOCT and validated with a micrometer after harvesting the heart. A total of 38 point lesions were created in the LA of 5 living swine with the integrated PSOCT-RFA catheter using standard clinical RFA procedures. For all lesions with analyzable PSOCT images, lesion transmurality was assessed with a sensitivity of 89% (17 of 19 tested positive) and a specificity of 100% (5 of 5 tested negative) using the quantitative transmural criteria. This is the first report of using PSOCT to assess LA RFA lesion transmurality in vivo. The results indicate that PSOCT may potentially provide direct real-time feedback for LA wall thickness and lesion transmurality.

## Introduction

Atrial fibrillation (AF) is the most common sustained arrhythmia in the western world^1^. With the aging population, it is predicted that 12–16 million people will be affected by AF by 2050 in the United States^2^. Radiofrequency (RF) catheter ablation (RFA) within the left atrium (LA) has become the standard treatment for AF^3^. It is estimated that there are 75,000 ablations performed for AF in the United State per year^4^, and the number is growing rapidly^5,6^. Single-lesion transmurality is critical to the efficacy of the procedure^7^. Contact force (CF)-sensitive (CFS) catheters have been widely adopted to monitor catheter-tissue CF in real time to improve lesion transmurality and outcomes^8^. However, with the guidance of CF and other indirect monitoring parameters (e.g., temperature and impedance), AF recurrence remains greater than 20% at 1-year-follow-up^9^. Additionally, AF RFA procedures are still associated with a significant risk of complications, such as collateral damage of surrounding tissues (e.g., phrenic nerve, aorta, esophagus), cardiac perforation, and microembolization^10,11^. Therefore, direct guidance based on visualization of cardiac tissue, including lesion formation information and cardiac wall thickness, in real time may provide the feedback necessary to titrate RF energy to improve lesion transmurality and procedure safety.

Many technologies are being studied to monitor lesion formation and assess lesion transmurality. For example, a novel ablation catheter has been developed to estimate lesion formation based on local impedance change^12–15^, and has been combined with CF to improve lesion prediction^16^. Though, more studies are needed to investigate the effectiveness of this technique. Magnetic resonance imaging (MRI) has been demonstrated to guide catheter-tissue contact^17–20^, monitor lesion formation in real-time^19,20^, and assess lesions size post-ablation^17,18,21,22^. However, MRI has limited resolution and requires expensive specialized equipment. Recently, near-field ultrasound^23,24^, opto-acoustics^25–27^, and near-infrared spectroscopy (NIRS)^28–31^, have been integrated with RFA catheters to monitor lesion formation in real time based on changes in ultrasound reflectivity, optical absorption, and absorption and scattering, respectively. The investigation of these numerous potential solutions confirms the urgent need for real-time direct lesion quality assessment for LA RFA procedures.

Optical coherence tomography (OCT) is a high-resolution noninvasive real-time imaging technique that is well suited for endoscopic imaging^32^. Early OCT studies have demonstrated that OCT imaging is able to visualize laser ablation of biological tissues^33,34^. We have demonstrated that OCT can provide tissue substrate information and differentiate RFA lesions from viable myocardial tissue^35,36^. Polarization sensitive-OCT (PSOCT) provides stronger contrast for cardiac RFA lesion formation compared to conventional backscatter OCT, based on the inherent change of myocardial birefringence during RFA^37^. Birefringence is an optical property of well-aligned tissue (e.g., muscle, collagen, and bone) and has been used to evaluate thermal damage^38,39^. We and others have also demonstrated that PSOCT can be integrated with an RFA catheter^40^ to confirm lesion formation^41^ and qualitatively monitor RF lesion transmurality in the right atrium in real time during a percutaneous RFA procedure^42^.

In this study, we prototyped an integrated PSOCT-RFA catheter that enabled PSOCT monitoring of percutaneous LA RFA procedures, which required crossing the atrial septum. Quantitative transmural lesion criteria were established with a well-controlled ex vivo experiment based on tissue birefringence monitored with PSOCT. The wall thickness and lesion transmurality monitoring capabilities of the integrated PSOCT-RFA catheter were evaluated in vivo in LA RFA procedures in healthy living swine.

## Results

### Integrated PSOCT-RFA catheter prototype

**Figure 1:**
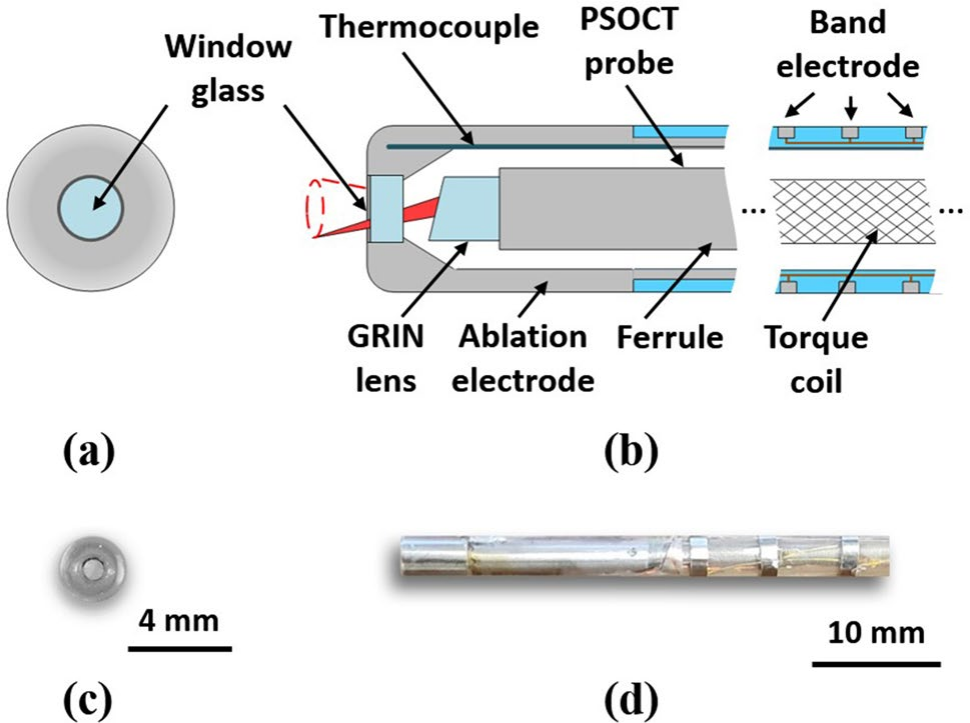
Diagrams and photographs of integrated PSOCT-RFA catheter tip. (**a**, **b**) are schematic drawings of the integrated catheter tip in front and cross-section views; (**c**, **d**) are photographs of the integrated catheter tip in front and side views.

An 8-Fr integrated PSOCT-RFA catheter was designed and prototyped, as shown in Fig. 1. A 4-mm long stainless-steel ablation electrode was designed with a thermocouple housed inside the distal end to measure temperature. It also accommodates a forward-imaging PSOCT probe in the central lumen and a 1-mm diameter window glass at the tip to allow for imaging. It was validated in previous work that the implementation of the window glass on the ablation electrode does not impact the ablation function^40^. The PSOCT probe was connected to the PSOCT imaging system and mounted on a rotary joint. The PSOCT laser beam focuses with an angle relative to the longitudinal direction, so the tissue was imaged with a 0.7-mm diameter scanning cone when the probe was rotated^40^. The lateral resolution is 17 µm. To enable electrogram recordings and 3D mapping (Ensite Velocity, St. Jude Medical, USA) compatibility, 3 band electrodes were included on the catheter proximal to the ablation electrode with a spacing of 3 mm, the functionality of which was validated by in vivoexperiments (experiment and results are described in the supplementary material). During in vivo experiments, the catheter was steered with an 8.5-Fr steering sheath (Agilis NxT Steerable introducer, St. Jude Medical, USA).

### Ex vivo experiment and quantitative transmural lesion criteria

**Figure 2:**
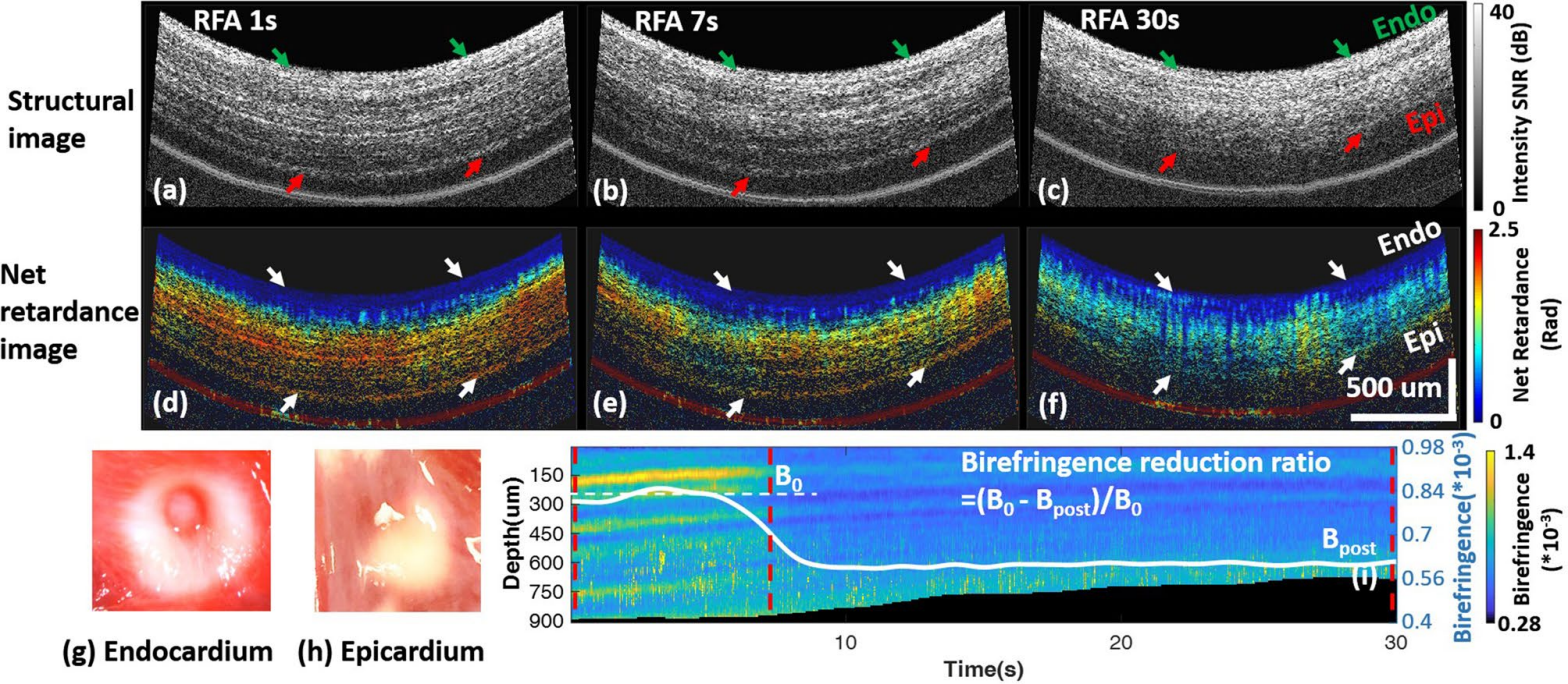
Ex vivo transmural lesion example at the posterior wall monitored with PSOCT. (**a**–**c**) and (**d**–**f**) are the structure images and net retardance images, respectively, of the lesion at 1 s, 7 s, and 30 s of ablation; (**g**, **h**) are photos of the TTC stained lesion on the endocardium and epicardium; (**i**) shows the cross-A-line averaged tissue birefringence change and the averaged tissue birefringence change (white curve). *Myo* Myocardium, *Extra* extra-myocardial tissue, *Endo* endocardium, *Epi* epicardium.

An example of a transmural lesion at the posterior LA wall monitored by PSOCT is shown in Fig. 2. Structural and net retardance images of the tissue at three time points during ablation are shown in panels (a–f). At the start of ablation (Fig. 2a,d), the myocardium has high birefringence, as indicated by the yellow and red color in the net retardance images. Tissue birefringence was high with much variability at different depths (Fig. 2i). As a lesion is created, proteins are denatured, strongly reducing tissue birefringence, and the net retardance images become more homogeneously blue (Fig. 2f). Tissue birefringence decreases and becomes more homogenous through all depths at 9 s, indicating the transmural change caused by RFA. The birefringence reduction ratio reached 30% at 9 s and remained stable. TTC staining confirmed lesion transmurality (Fig. 2g,h).

Birefringence reduction ratios of all 20 ex vivo lesions were evaluated and grouped based on lesion transmurality from TTC staining. Birefringence reduction ratio for transmural lesions (n = 15) and non-transmural lesions (n = 5) fell in two separate normal distribution groups (Shapiro–Wilk test). A reduction ratio ≥ 6.8% was chosen as the quantitative threshold to classify a lesion as transmural, because it correctly separates 99% of ex vivo data (Fig. 6a).

### In vivo experiment

**Figure 3:**
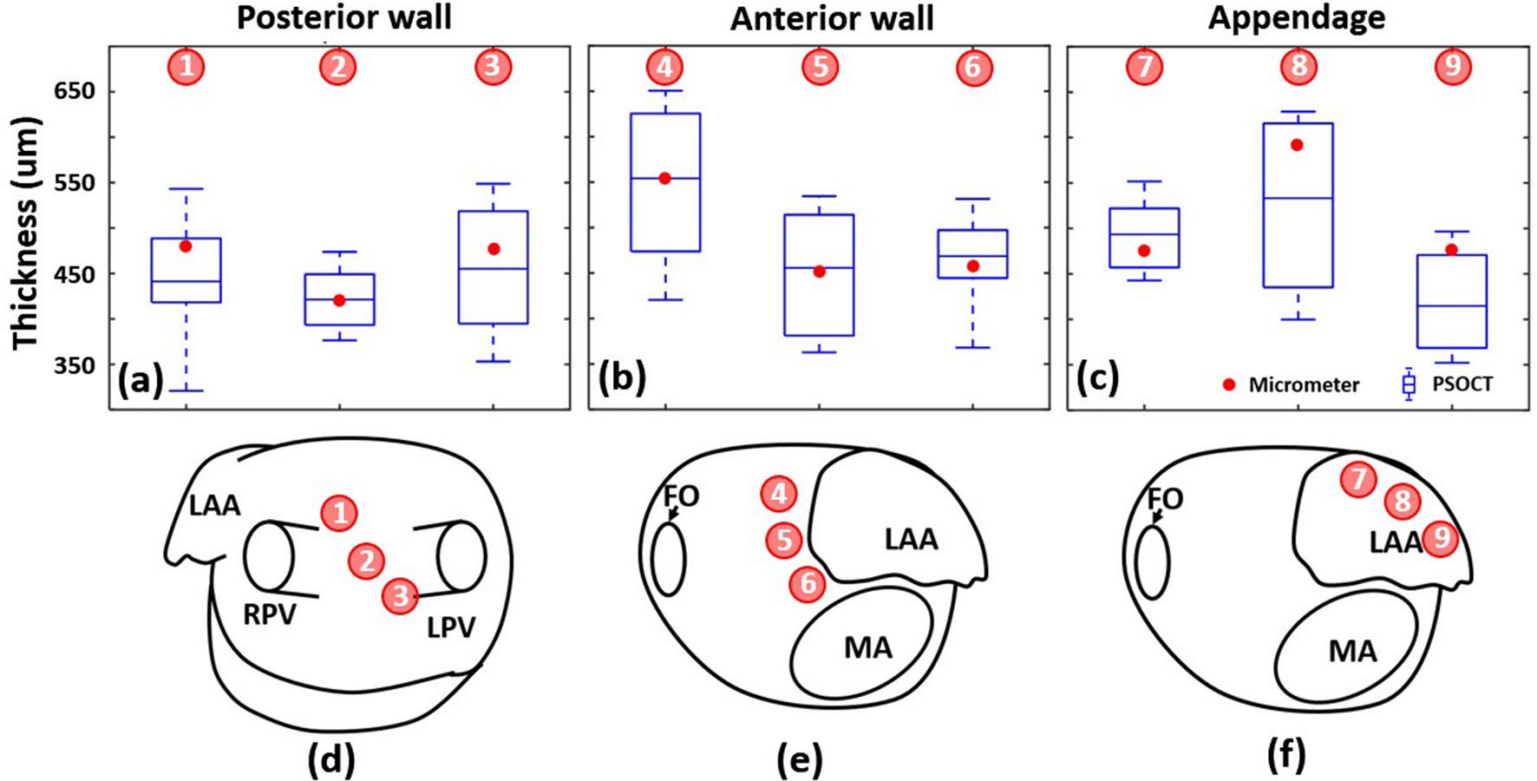
Cardiac LA wall thickness measurement validation. (**a**–**c**) are the comparison of wall thickness measured with PSOCT images (Whiskers, maximum and minimum; box, standard deviation; line, mean) and micrometer (red dots). (**d**–**f**) are maps of the anterior and posterior LA wall with locations noted for the wall thickness measurements. *FO* Foreman ovalis, *LAA* Left atrium appendage, *MA* Mitral annulus, *RPV* Right pulmonary vein, *LPV* Left pulmonary vein.

In vivo cardiac wall thickness measurements of 9 locations at the posterior and anterior wall, and LAA are shown in Fig. 3, with the locations noted in panels (d–f). As described above, PSOCT measures wall thickness as a range, therefore it is represented by the box and whisker plot in panels (a–c). Most of the wall thicknesses validated with the micrometer (8/9, 88.9%) fell within the boxes, i.e. the standard deviation of PSOCT measurements.

**Figure 4:**
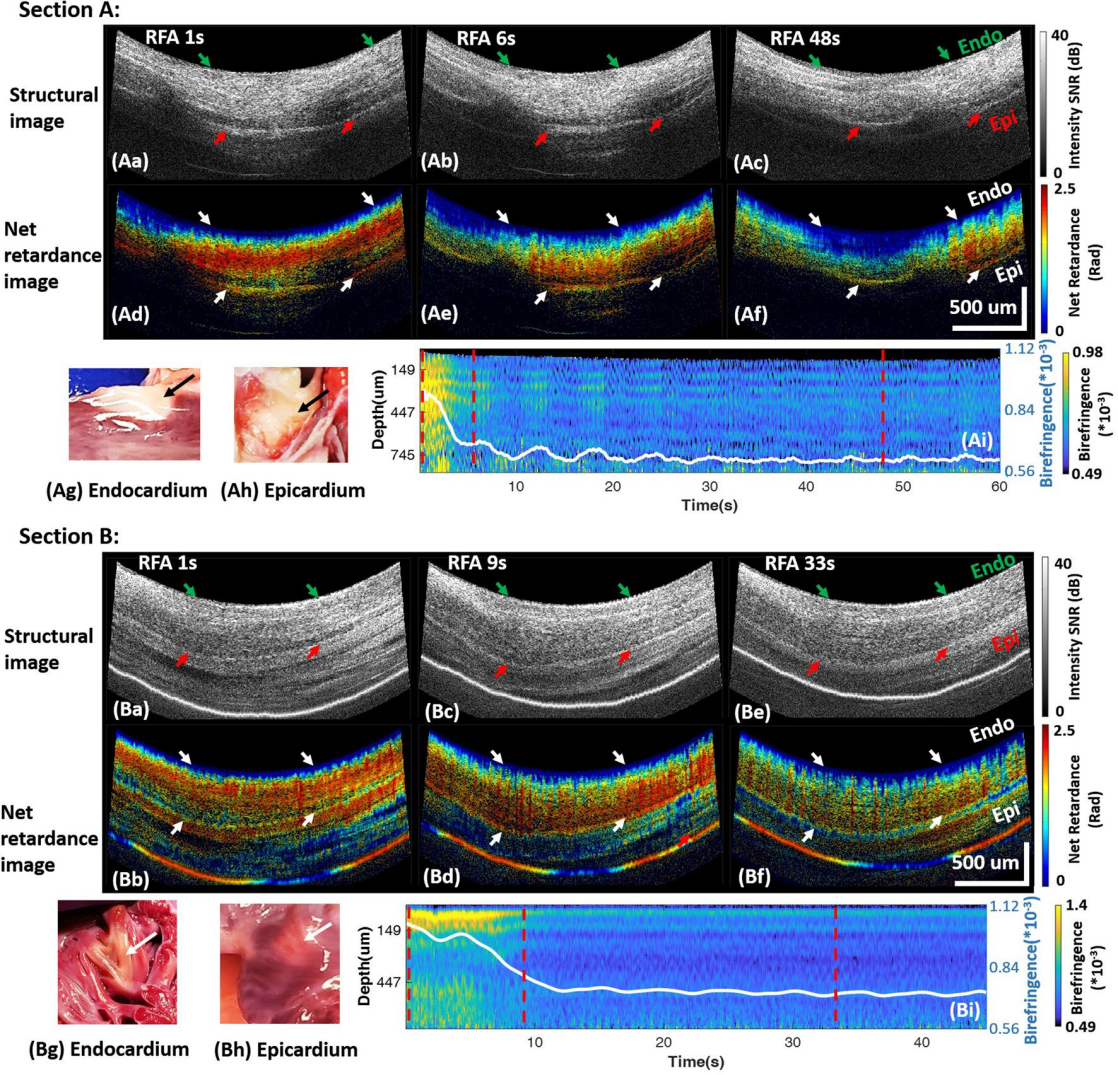
Transmural lesions at the LPV (Section A) and LAA (Section B). For both sections A and B, panels (**a**–**c**) and (**d**–**f**) are the structure images and net retardance images of the lesion at three time points during ablation, respectively; panels (**g**, **h**) are photos of the TTC stained lesion on the endocardium and epicardium; (**i**) shows the cross-A-line averaged tissue birefringence change and the averaged tissue birefringence change. Videos are provided as supplementary video v4 and v5.

In vivo PSOCT monitoring of a transmural lesion created near the left pulmonary vein (LPV) is shown in Fig. 4 (Section A). Similar to Fig. 2, the disappearance of yellow and red band structure in the net retardance image as time progresses during ablation (Ad–Af)) indicates the formation of a lesion. The averaged birefringence across A-lines reduced through the cardiac wall over time (Ai). The averaged birefringence trace shows that tissue birefringence reduced quickly between 0 and 7 s, and the birefringence reduction ratio reached 15.8% by 30 s and remained stable, which met the quantitative transmural lesion criteria. The result agreed with TTC lesion transmurality assessment shown in Fig. 4Ag, Ah. A transmural lesion created in the left atrium appendage (LAA) is shown in Fig. 4 (Section B). In this example, the cardiac wall structure was more complicated. There was more extra-myocardial tissue observed beyond the myocardial wall. Net retardance of the myocardial wall also diminished (Fig. 4Bd–Bf) and the birefringence reduction ratio reached 16.7% at 17 s and remained stable (Fig. 4Bi), indicating a transmural lesion. The result agreed with TTC staining lesion transmurality analysis (Fig. 4Bg,Bh).

**Figure 5:**
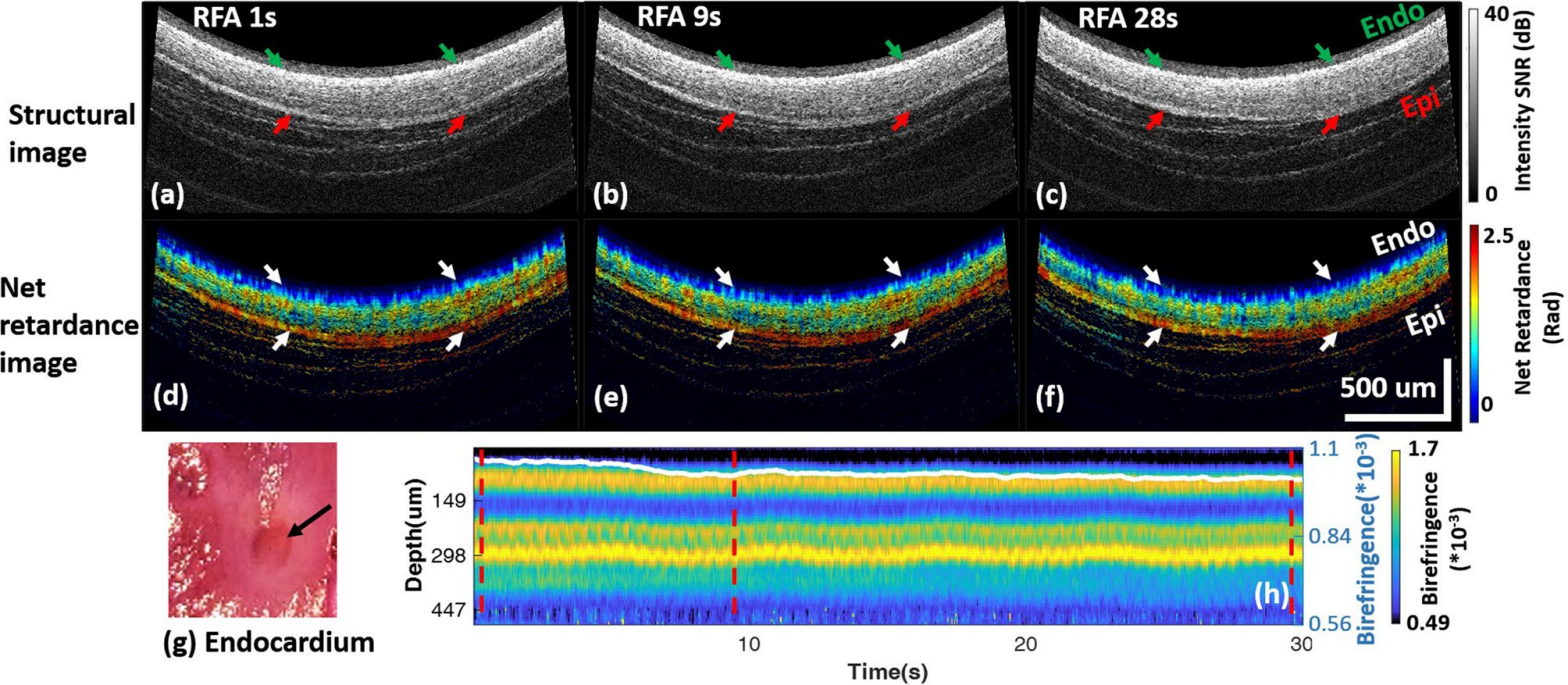
A non-transmural lesion around the RPV. (**a**–**c**) and (**d**–**f**) are the structure images and net retardance images of the lesion at 1 s, 8 s, and 29 s of ablation, respectively; (**g**) is a photo of the TTC stained lesion on the endocardium; (**h**) shows the cross-A-line averaged tissue birefringence change and the averaged tissue birefringence change.

An example of a non-transmural lesion at the right pulmonary vein (RPV) ostia monitored with PSOCT is shown in Fig. 5. The net retardance images at different time points during ablation remained stable, which can be visualized by the sustained yellow and red structures in (d–f). The averaged birefringence across A-lines (panel h) did not reduce through the cardiac wall over time as seen in Fig. 4Ai,Bi. The birefringence reduction ratio of this lesion was 4.4% and did not meet the quantitative PSOCT criterion for a transmural lesion. This agreed with the TTC staining, shown in panel (g), which does not indicate the presence of a transmural lesion.

Immediately before each ablation attempt, cardiac wall thickness was measured with PSOCT at the ablation site (Supplementary Fig. S6). The measured posterior wall and LAA thickness averaged 482 ± 273 µm and 475 ± 214 µm, respectively. Because of the trabeculated structure of LAA, the catheter was usually wedged between trabeculae and only the thinner wall was measured. The roof and the wall around mitral annulus were the thickest areas measured in the LA, where the measured thickness averaged 626 ± 55 µm, and half of the sampled locations were too thick to be imaged transmurally. In some pigs, the wall thickness at the middle inferior posterior wall and the junction of LPV and posterior wall were beyond the imaging range. Lesions at locations beyond the imaging range were not analyzed for wall thickness. Pre-ablation wall thickness could not be confirmed with the micrometer because of thickness changes due to ablation; however, for all locations with transmural images, the thickness agreed with the corresponding thickness measured in the validation experiment (Fig. 3). This shows the consistency of PSOCT cardiac wall thickness measurement.

**Figure 6:**
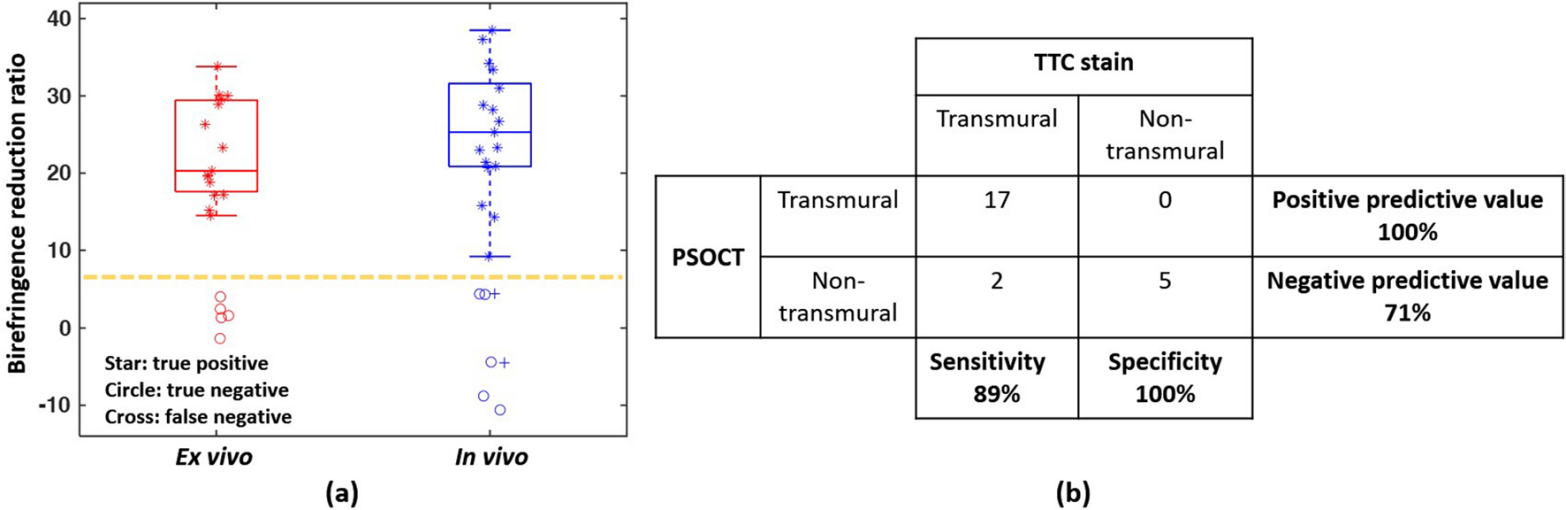
Summary of all analyzable data. (**a**) Birefringence reduction ratio comparison for all ex vivo and analyzable in vivo data; (**b**) confusion matrix for all analyzable in vivo data.

In sum, there were 38 RF lesions created in the LA of five pigs and examined with TTC for transmurality. Besides impedance drop and temperature rise, analysis of the electrogram amplitude at lesion sites showed that the mean electrogram amplitude after RF ablation (1.97 mV) was significantly less than that before RF ablation (2.40 mV, *p* < 0.05, n = 13, Students paired T-test). All recorded PSOCT datasets were classified as analyzable data (24/38, 63%) (Supplementary Fig. S3) or non-analyzable data (14/38, 37%). Of all analyzable PSOCT datasets, 17 lesions were identified as transmural, and 7 were identified as non-transmural, in which 5 agreed with TTC and 2 did not (Fig. 6b). For the 17 transmural lesions, the transmural lesion criterion was achieved by 19.5 ± 17.5 s. Accordingly, in this study, the sensitivity and specificity of PSOCT monitoring lesion transmurality are 89% and 100%, respectively. The positive predictive value is 100%, and negative predictive value is 71%, as shown in Fig. 6b. The area under the receiver operating characteristic curve is 0.97.

Of the 14 (37%) non-analyzable datasets, 11 (29%) were rejected due to unstable PSOCT images and the other 3 were rejected due to tissue thickness beyond the field of view for PSOCT. Considering all of the lesion attempts in this study, TTC staining identified non-transmural lesions in 8/14 (57%) in the non-analyzable dataset, but only 5/24 (21%) in the analyzable dataset (Chi-square test, *p* = 0.02, α = 0.05). This suggests that when PSOCT images were unstable, so was the ability to create transmural lesions.

## Discussion

Although RFA has become one of the primary treatments for AF, recurrence of AF remains high^3,9^. Studies have confirmed that non-transmural RFA lesions are an important factor leading to recurrance of AF^7^. Accordingly, many efforts have been attempted to provide lesion quality guidance to improve RFA procedure outcomes. In this study, we designed and fabricated an integrated PSOCT-RFA catheter and validated its monitoring functionality in LA RFA procedures in healthy living swine. Results demonstrated that PSOCT structural images enabled the visualization of cardiac wall structure and the measurement of cardiac wall thickness with high-resolution, which is critical for lesion quality estimation and for minimizing collateral damage. Myocardium birefringence, as a tissue optical property, can be used to evaluate the thermal injury of the myocardium caused by RF energy^43^. In this work, we demonstrated that myocardium birefringence reduction can be used to quantitatively assess lesion transmurality with a high sensitivity of 89% and specificity of 100% in analyzable PSOCT datasets.

**Figure 7:**
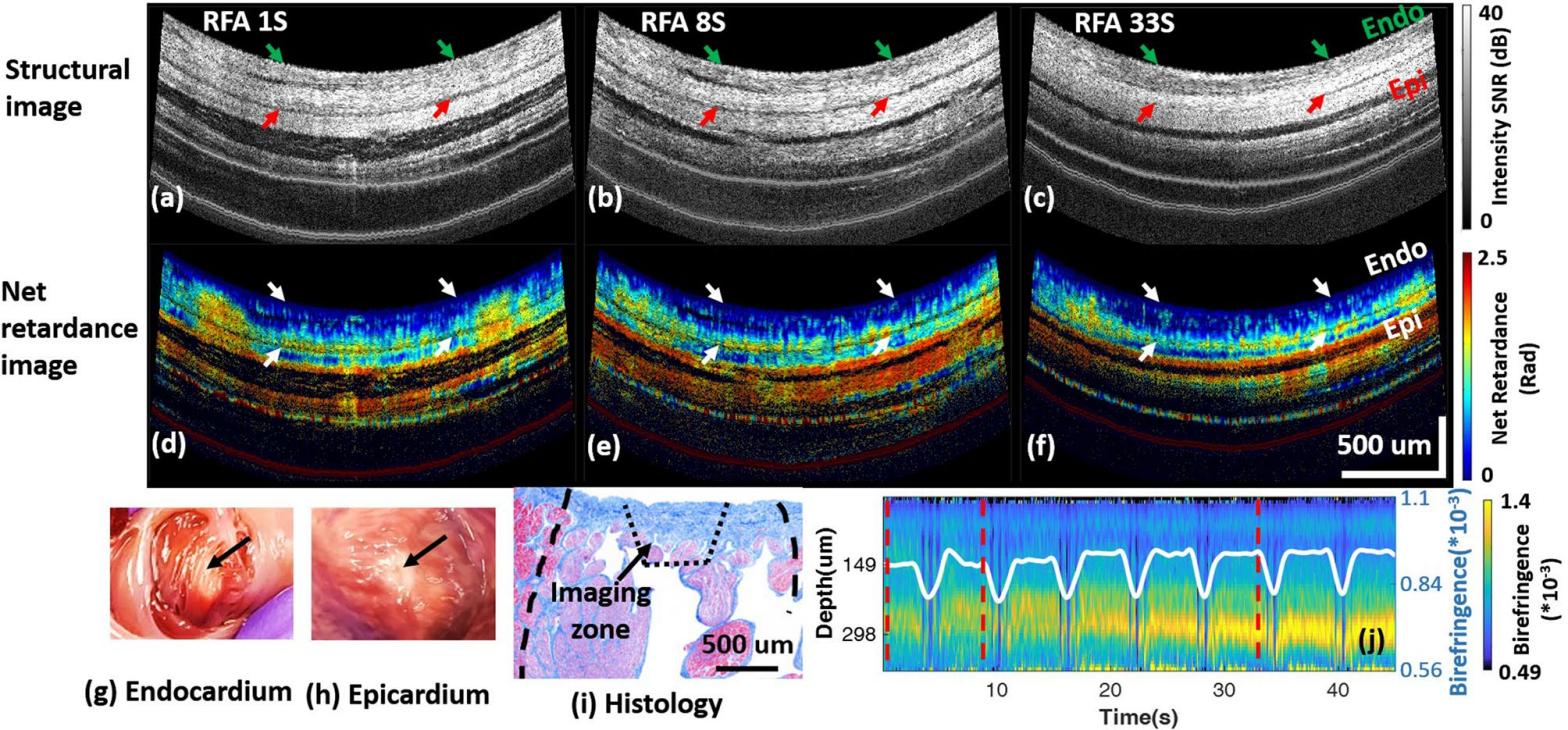
A false negative lesion at LAA. (**a**–**c**) and (**d**–**f**) are the structure images and net retardance images of the lesion at 1 s, 8 s, and 33 s of ablation, respectively; (**g**, **f**) are photos of the TTC stained lesion on the endocardium and epicardium; (**i**) histology of lesion section with trichrome staining; (**j**) shows the cross-A-line averaged tissue birefringence change and the averaged tissue birefringence change.

Two lesions were identified as transmural by TTC staining, but categorized as non-transmural by PSOCT images. Both of these false negatives were in the superior LAA, and showed similar tissue structure and birefringence change, one of which is shown in Fig. 7. Neither the structure images, net retardance images, nor the quantified tissue birefringence exhibited the changes characteristic of a transmural lesion. However, TTC staining showed that it was transmural (Fig. 7g,h). Histology of this lesion cross-section (Fig. 7i) indicated that part of the cardiac wall that would have been within the PSOCT field of view is mainly composed of collagen, not myocardium. Collagen has different birefringence from myocardium^44^ and denatures differently with thermal damage^43^. This may be the reason why tissue birefringence measured at this lesion site was different from other sites and was therefore mis-classified by PSOCT. To increase the robustness of PSOCT lesion transmurality monitoring, more experiments should be done to include various LA wall tissue types to develop and refine classification criteria.

Unstable PSOCT images were the primary cause of the non-analyzable datasets (11/38, 28.9%). This is likely because all lesions in this study were created using standard clinical practice without any guidance on catheter-tissue orientation to obtain stable PSOCT images. In the future, using PSOCT to guide catheter placement may improve catheter-tissue stability and, thus, lesion formation. Furthermore, the integrated PSOCT-RFA catheter lacked steering and was guided by a steerable sheath, which may have increased the difficulty to achieve and maintain perpendicular catheter-tissue orientation. A future, steerable integrated PSOCT-RFA catheter may facilitate image stability. The current integrated PSOCT-RFA catheter is forward-viewing, which limits tissue monitoring while in angled contact. Most operators utilize an angled catheter-tissue orientation in the LA at some locations, such as the LA septum. Therefore, future PSOCT-RFA catheter designs with multiple viewing angles may enable lesion transmurality monitoring under more catheter-tissue orientations.

PSOCT was able to image full tissue thickness for most lesion transmurality assessments in the LA (27/38, 71.1%, Supplementary Fig. S6). However, there are thick wall areas where PSOCT imaging depth is insufficient. To overcome this limitation, a model was previously developed to estimate lesion depth with the measured denature time of the tissue within the depth of view^45^. Another approach under investigation, NIRS, detects RFA lesion depth as deep as 4 mm^31^, but it doesn’t provide detailed tissue structure information. More effort is warranted to overcome this limitation in the future.

The integrated PSOCT-RFA catheter used in this study lacks CFS and irrigation. Lesions created with CFS and irrigated catheters may form differently^8,46^. However, because the principle of monitoring lesion transmurality with PSOCT is not a function of CF or irrigation, we expect the monitoring functionality will work similarly in future studies which include CFS and irrigation.

Through direct tissue feedback and monitoring of lesion transmurality, PSOCT has the potential to improve efficacy of lesion creation and therefore ablation success rates. Furthermore, it could reduce excess RF energy delivered and therefore may improve procedure safety. We observed in vivo that for thin LA walls (< 1 mm) with stable images, birefringence was reduced to its lowest value by an average of 17.4 s, which is shorter than the duration of a standard ablation protocol. This suggests that on thin cardiac walls, more RF energy tends to be delivered than needed to create transmural lesions with current standard clinical RFA protocols, which potentially increases the risk of damaging the cardiac wall or collateral tissue. This further suggests that birefringence may potentially be used to titrate RF energy delivery for durable transmural lesion creation, as opposed to current practice which is to ablate for a fixed, predetermined time at a pre-determined power.

We noted that more non-transmural lesions were created when unstable PSOCT images were observed. This implies that unstable catheter-tissue contact (indicated by unstable images) can lead to poor quality lesion creation. PSOCT is capable of confirming catheter-tissue apposition^40,42^ and stability (supplementary material), so future studies may use this information to control catheter-tissue contact.

We report the first clinically relevant in vivo LA RFA lesion transmurality assessment with an integrated PSOCT-RFA catheter. LA wall thickness was measured accurately with PSOCT. Also, for the first time tissue birefringence reduction ratio was quantified during ablation, and lesion transmurality was ascertained with high sensitivity and specificity, which may potentially improve the outcome of LA RFA procedures. Further development and studies are warranted, including next-generation PSOCT-RFA catheters incorporating steering and irrigation, and measures to mitigate current limitations of imaging depth range and forward-only imaging. The results of this study are encouraging and justify such further development.

## Methods

### PSOCT imaging system

The PSOCT imaging system has a center wavelength of 1310 nm, an axial resolution of 10 µm, and a sensitivity of 100 dB^47^. The maximum penetration depth of PSOCT in living swine LA was about 1 mm. PSOCT scans a polarized laser beam over a sample to form images. A single depth scan is called an A-line, and multiple (1500) A-lines form a single image frame. For this experiment, raw image data were collected at 24 frames/s. Structural images from one channel were displayed at 6 frames/s in real-time for observation. In post processing, structural images, net retardance images, and birefringence images^48^ were calculated for systematic analysis (Matlab 2019a).

### Ex vivo experiment

To study the lesion monitoring function of PSOCT in a well-controlled experiment, ex vivo experiments were performed in LA dissected from 3 fresh swine hearts with a mass of 500 ± 60 g. Tissue was submerged in a 37 °C circulated phosphate-buffered saline (PBS) bath. RFA lesions were created using the integrated PSOCT-RFA catheter and a standard RF generator (Maestro 3000, Boston Scientific, USA, temperature mode, power 40 W, temperature 65 °C) with a range of ablation times (5, 15, 30, 45 s). PSOCT images were recorded during ablation. The tissue was subsequently stained with 1% triphenyl tetrazolium chloride (TTC) PBS solution at 37 °C for 30–60 min to evaluate lesion transmurality. Lesions stained white on both endocardium and epicardium were identified as transmural.

Net retardance, the accumulated effect of birefringence through depth, was used as a qualitative evaluation of lesion formation. Birefringence through depth was calculated by averaging tissue birefringence across A-lines within an image and was used to quantitatively evaluate lesion formation at different depths. Frame-wise averaged birefringence in the myocardial wall was calculated for each timestamp. The averaged birefringence at the start of ablation was defined as B_0_ and the lowest birefringence that remained stable (≥ 5 s) was defined as B_post_ (Fig. 2i). Birefringence reduction ratio, (B_0_ − B_post_)/B_0_, was calculated to quantitatively evaluate the transmural thermal damage induced with RF energy for all lesions.

### In vivo model

In vivo experiments were performed in the LA of 5 healthy pigs weighing 66 ± 4 kg at the Atrial Fibrillation Innovation Center (Cleveland Clinic, USA). The experiment was performed in accordance with a protocol approved by the Institutional Animal Care and Use Committee of the Cleveland Clinic and complied with the ARRIVE guidelines. Under anesthesia, the LA was accessed percutaneously by transseptal puncture (Brockenbrough needle, St. Jude Medical, USA) with the guidance of biplane fluoroscopy and intracardiac echocardiography (8-Fr AcuNav, Johnson & Johnson Medical, USA).

### Wall thickness measurement validation

To validate in vivo wall thickness measurements, PSOCT images were recorded at three different locations within each of three areas in a living swine: the anterior wall, inferior wall, and LA appendage (LAA). All locations were recorded on the 3D map. After harvesting the heart, the wall thickness at each location was measured with a micrometer, which applies the same pressure on the tissue for every measurement (Mitutoyo Corporation, Japan, resolution 0.01″, contact force 5 N) three times on the fresh tissue. The averaged micrometer measurements were used as the gold standard to validate the PSOCT measurement in vivo.

**Figure 8:**
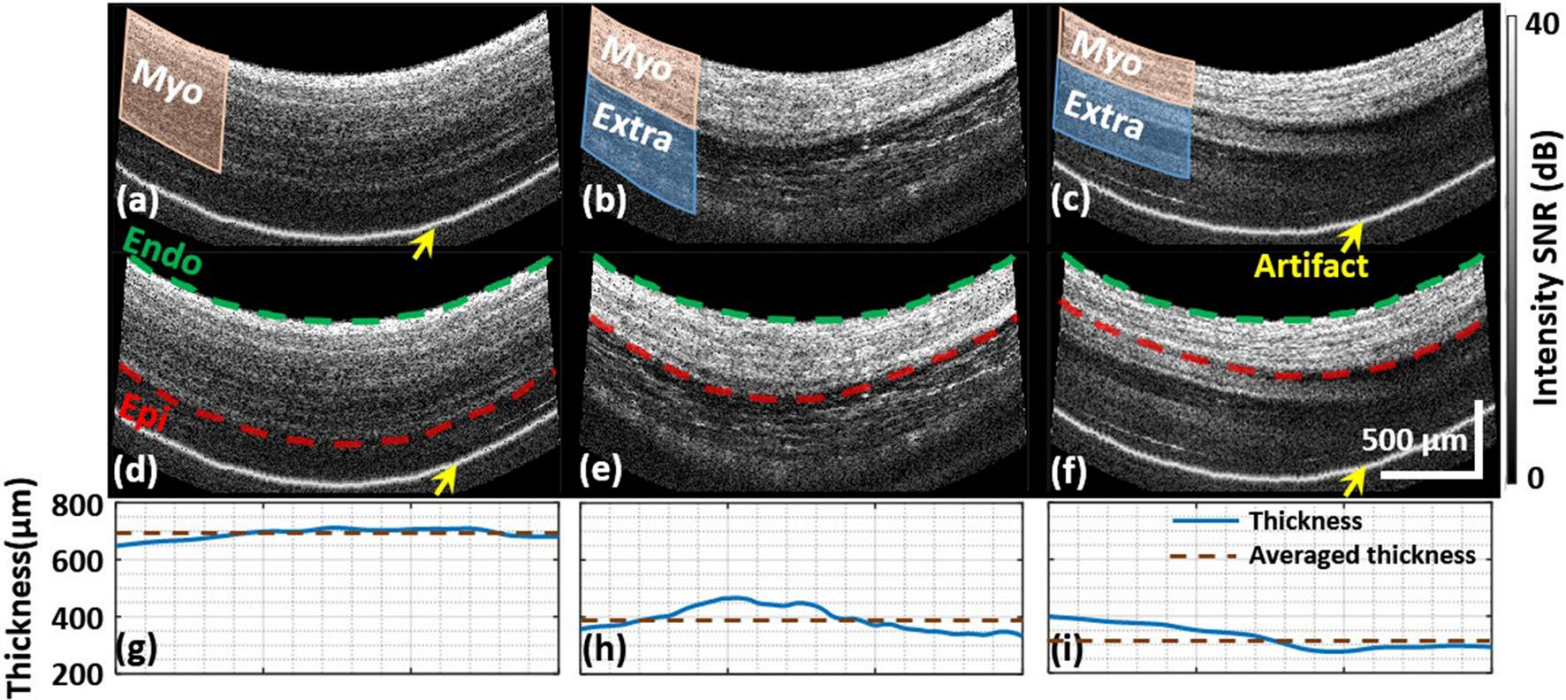
Illustration of the segmentation of myocardial wall. (**a**–**c**) are examples of structural images with different extra-myocardial tissue; (**d**–**f**) are the segmented endocardium and epicardium of (**a**–**c**), respectively; (**g**–**i**) are the wall thickness and averaged thickness of (**a**–**c**), respectively. The yellow arrows indicate reflection artifacts that originate within the imaging probe, which varies from probe to probe.

PSOCT image streams were recorded over several heartbeats at each location. Endocardium and epicardium were segmented manually with structural images during diastole using the myocardium and adipose tissue^35^ texture difference as shown in Fig. 8a,b. In some cases, the extra-myocardial tissue’s texture (e.g., pericardium) is similar to myocardium and difficult to differentiate in still images. However, it was clearly differentiable from myocardium as the tissues move during heartbeats (Fig. 8c, Supplementary video v1), and tissue movement was observed for accurate segmentation. Wall thickness was calculated as the distance between the endocardium and epicardium, and the averaged thickness was used as the thickness for each location, as shown in Fig. 8d–i.

### RFA lesion transmurality monitoring validation

To validate in vivo RFA lesion transmurality monitoring with PSOCT in the LA, lesions were created with the integrated PSOCT-RFA catheter and the same RF generator (temperature mode, power 30–40 W, temperature 55–65 °C, 30–60 s). In order to confirm that RF energy was delivered as is done clinically, tissue impedance drop and temperature increase were monitored during ablation, and reduction of electrogram amplitude was recorded before and after ablation (in three of the animals). To enable examination of the transmurality of single lesions by pathology after heart excision, lesions were ablated sparsely in the LA with locations separated and recorded on the 3D map. PSOCT image recording was initiated and stopped manually at the same time as ablation for each lesion. After harvesting the hearts, lesion transmurality was assessed using TTC staining as described above for the ex vivo experiments. Identified lesions were matched to corresponding PSOCT images by referring to the locations on the 3D map.

### PSOCT data analysis for assessment of lesion transmurality

PSOCT data quality is highest when the catheter tip is in perpendicular contact with the heart wall, displacing blood between the tissue and the glass window. However, when imaging within the living heart, continuous perpendicular contact is not always achieved. This leads to variability in the quality of image streams. All PSOCT datasets were first evaluated for image stability (details in the supplementary material). For this study, all stable datasets with transmural images (the full thickness of the atrial wall was visible) were classified as analyzable data. Net retardance images and birefringence reduction ratio were evaluated in the same way as described above to assess lesion transmurality for all analyzable datasets.

### Ethics declarations

The living swine experiment reported in this work was performed in accordance with a protocol approved by the Institutional Animal Care and Use Committee of the Cleveland Clinic and complied with the ARRIVE guidelines. No human subjects were involved in this work.

## Supplementary Information

Supplementary Video 1.

Supplementary Video 2.

Supplementary Video 3.

Supplementary Video 4.

Supplementary Video 5.

Supplementary Information 1.

## Data Availability

The datasets generated during the current study are available from the corresponding author upon reasonable request.
