# Author Correction: Polarization-sensitive optical coherence tomography monitoring of percutaneous radiofrequency ablation in left atrium of living swine

**DOI:** 10.1038/s41598-022-07500-0

**Published:** 2022-03-01

**Authors:** Xiaowei Zhao, Ohad Ziv, Reza Mohammadpour, Benjamin Crosby, Walter J. Hoyt, Michael W. Jenkins, Christopher Snyder, Christine Hendon, Kenneth R. Laurita, Andrew M. Rollins

**Affiliations:** 1 Department of Biomedical Engineering, Case Western Reserve University, Cleveland, OH USA; 2 School of Medicine, Case Western Reserve University, Cleveland, OH USA; 3 Heart and Vascular Research Center, MetroHealth Medical Center, Cleveland, OH USA; 4 Department of Chemistry, Case Western Reserve University, Cleveland, OH USA; 5 The Congenital Heart Collaborative, Rainbow Babies and Children’s Hospital, Cleveland, OH USA; 6 Department of Pediatrics, Case Western Reserve University, Cleveland, OH USA; 7 Case Western Reserve University, Cleveland, OH USA; 8 Department of Electrical Engineering, Columbia University, New York, NY USA; 9 Department of Pediatrics, Ochsner Health, New Orleans, LA USA

Correction to: *Scientific Reports* https://doi.org/10.1038/s41598-021-03724-8, published online 21 December 2021

The Acknowledgements section in the original version of this Article was incomplete.

“This research was supported by National Institutes of Health (NIH) (R01HL149369, R21HL129174, R01HL126747, UH54HL119810); Case-Coulter Translational Research Partnership; CWRU Technology and Validation Start-Up Fund Program; China Scholarship Council. We acknowledge Dr. David Von Wagoner and the Atrial Fibrillation Innovation Center (Cleveland Clinic) for the help with this animal experiment.”

now reads:

“This research was supported by National Institutes of Health (NIH) (R01HL149369, R21HL129174, R01HL126747, UH54HL119810); Case-Coulter Translational Research Partnership; CWRU Technology and Validation Start-Up Fund Program; China Scholarship Council. We acknowledge Dr. David Von Wagoner and the Atrial Fibrillation Innovation Center (Cleveland Clinic) for the help with this animal experiment. The content is solely the responsibility of the authors and does not necessarily represent the official views of the National Institutes of Health.”

The original Article has been corrected.

