# Supplementary material for: Polarization-sensitive optical coherence tomography monitoring of percutaneous radiofrequency ablation in left atrium of living swine
Source: Sci Rep. 2021 Dec 21;11:24330. doi: 10.1038/s41598-021-03724-8 (PMC8692484; doi:10.1038/s41598-021-03724-8)
Supplement: Supplementary file 6 — Supplementary Information 1. [file 41598_2021_3724_MOESM6_ESM.docx]

1. **Polarization-sensitive optical coherence tomography (PSOCT) data analysis for lesion quality assessment**

**
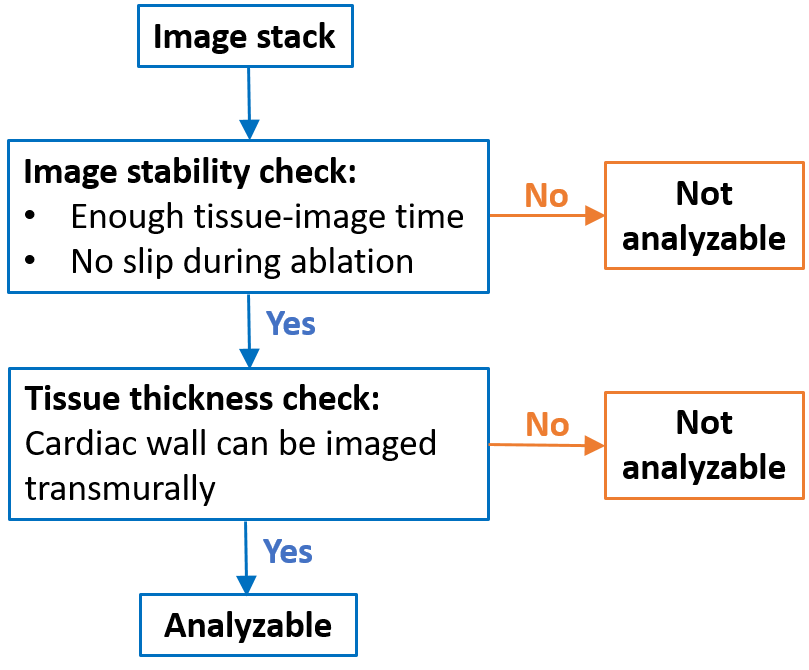
**

**Supplementary figure S1. Flow chart of classification of image sets as analyzable data and not-analyzable data**

PSOCT images the cardiac wall in front of the window glass by displacing blood between the cardiac wall and catheter tip. With stable and transmural cardiac wall images, wall thickness and lesion transmurality can be assessed. However, in this study, using standard clinical practices for left atrium (LA) radiofrequency ablation (RFA), this was not achieved every time, leading to variable PSOCT image quality. In this study, all PSOCT datasets wherein the catheter was in contact with the tissue for at least 70% of ablation time, and no slipping of the catheter against the tissue was detected during ablation were considered to be stable. Stable-image datasets with transmural images were classified as analyzable, as shown in Supplementary fig. S1.


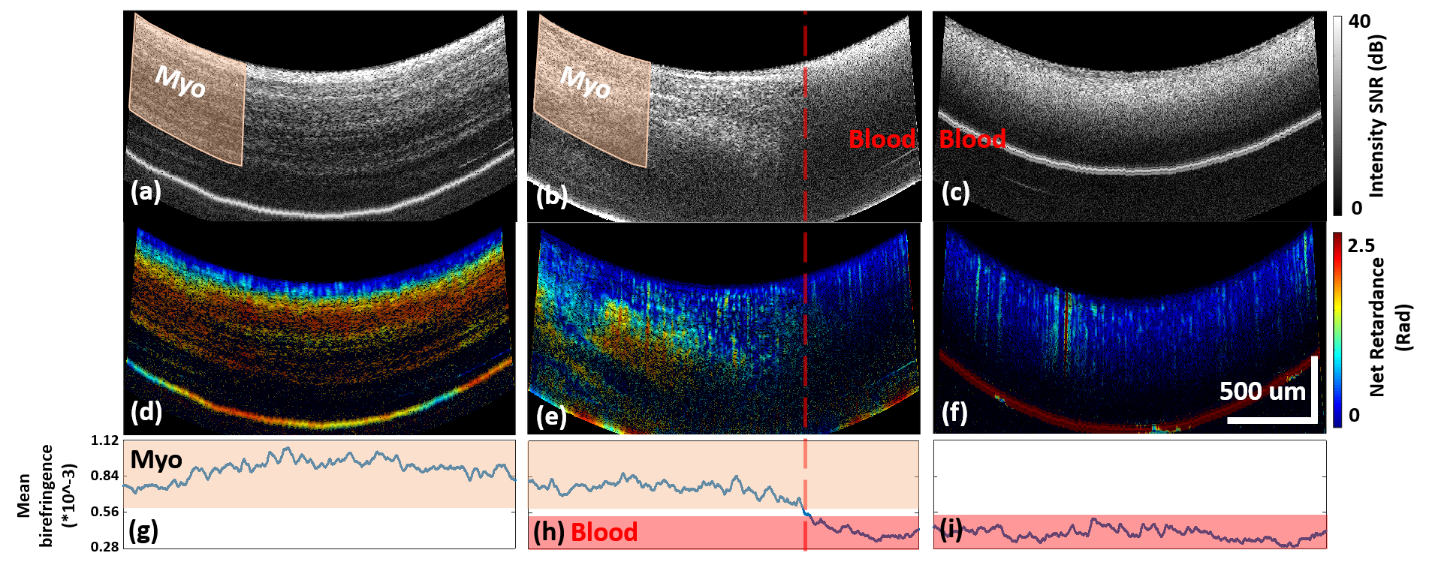


**Supplementary figure S2. Tissue-blood state analysis. (a) - (c) are examples of full-tissue, part-tissue, and full-blood images; (d) - (f) are the net retardance images of (a) - (c), respectively (g) - (h) are the averaged birefringence along A-lines for (a) – (c) respectively.**

1.1 Tissue-image time calculation

All image frames in a dataset were classified into full-tissue, part-tissue, and full-blood states based on the content in the image (supplementary fig S2 (a) – (c)). Different from the myocardium, blood is not birefringent. Therefore, averaged birefringence along A-lines was calculated to identify the tissue in one A-line. A-lines with average birefringence higher than 0.038 were identified as myocardium, as shown in supplementary fig. S2 (d) – (f). Tissue-image time was calculated as the total fraction of images displaying full-tissue and part-tissue.

1.2 Slip recognition

The correlation of the images at the same phase of sequential heartbeats was calculated for each dataset to evaluate the changes. Datasets that had an abrupt change in the correlation curve which corresponds to the loss of tissue in the field of view were considered to demonstrate that the catheter had slipped against the atrial wall during ablation.

- 1. Classification results and locations

As shown in supplementary fig. S3, all recorded PSOCT datasets were classified into analyzable datasets (24/38, 63%), and non-analyzable datasets (14/38, 37%). Non-analyzable datasets are labeled as unstable datasets (11/38, 29%, an example is provided as supplementary video 2) and stable but nontransmural image datasets (3/38, 8%, an example is provided as supplementary video 3) corresponding to the two classification steps in supplementary fig. S1.


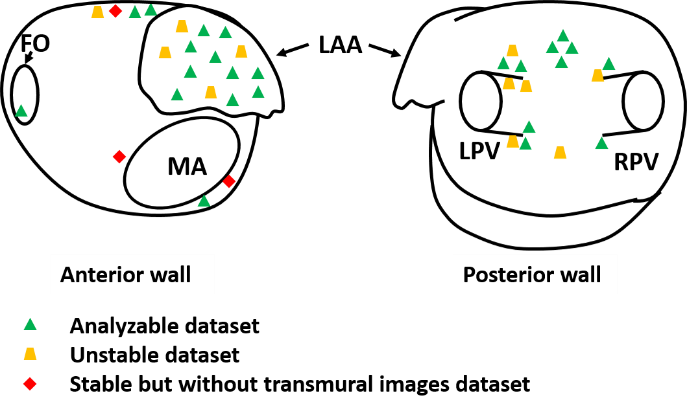


**Supplementary figure S3. PSOCT analyzable and non-analyzable classification map. Unstable datasets and datasets that were stable but without transmural images were classified as non-analyzable. LAA, left atrial appendage; LPV, left PV; RPV, right PV; MA, mitral annulus; FO, foraman ovale.**

1. **Heartbeat noise filter for birefringence change curve**

Previous research has shown that the birefringence of muscle changes during contraction and relaxation^1^. It was observed in this study that myocardium contraction and relaxation during the heartbeat introduced 1.5-Hz noise on the birefringence trace, which is the same frequency as the heartbeat. To remove this noise, a low pass filter with a cutoff frequency of 1 Hz was applied, as shown in supplementary fig. S4.


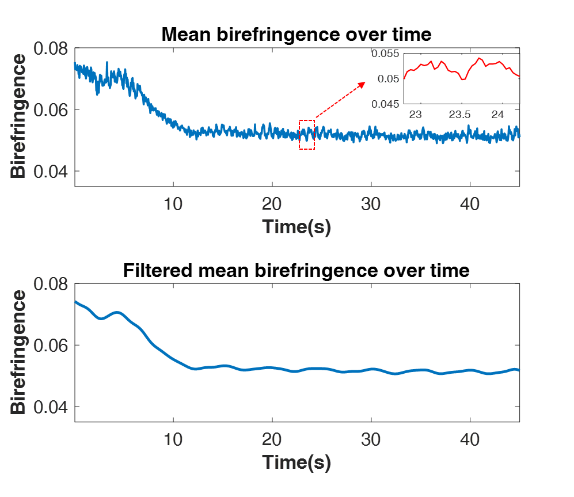


**Supplementary figure S4. An example of averaged birefringence curve heartbeat noise removal.**

1. **Electrogram and 3D mapping validation**

Clinical LA RFA procedures rely on the guidance of electrogram measurement and 3D mapping. Therefore, it was necessary to validate the electrogram and 3D mapping functionality of the integrated PSOCT-RFA catheter developed for this study. Electrograms were recorded near the atrioventricular (AV) node with the integrated PSOCT-RFA catheter and a standard mapping catheter (Decapolar, St Jude), and were compared to validate the electrogram mapping functionality of the integrated catheter. A 3D map of the LA and the pulmonary veins (PVs) was generated with the standard mapping catheter. The integrated catheter was then used to map the PVs without the assistance of the standard catheter. The two maps were compared to validate the 3D mapping functionality of the integrated catheter.

**
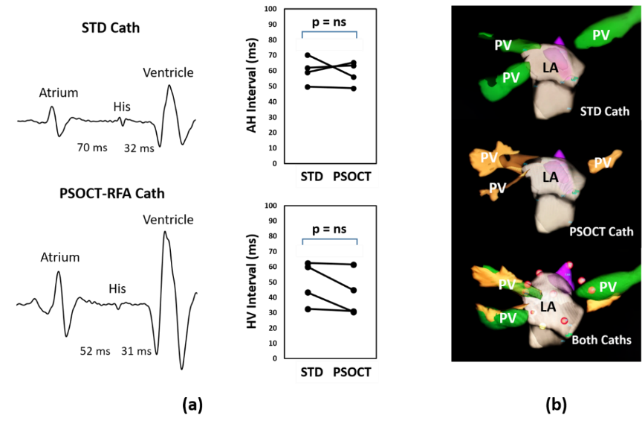
**

**Supplementary figure S5. Electrocardiogram (a) and 3D mapping function (b) validation. STD, standard mapping catheter; AH, atrial-his; HV, his-ventricle; PV, pulmonary vein.**

AV node electrograms measured with the integrated PSOCT-RFA catheter and a standard mapping catheter are similar, as shown in supplementary fig. S5 (a). In addition, the weak signal from His bundle is observed in both electrograms, which means the PSOCT-RFA catheter is suitable for recording electrograms. Atrial-His (AH) interval and His-ventricle (HV) interval were also similar for the electrograms measured with both catheters. The comparison of the PV 3D maps generated with the PSOCT-RFA catheter and the standard catheter is shown in panel (b). Because the standard mapping catheter has 5 pairs of mapping electrodes and the PSOCT-RFA catheter has 2, the map generated with the integrated PSOCT-RFA catheter doesn’t have dense data points. But they are at the same location of LA and overlapped with each other. These validation results demonstrate that the tip and band electrodes of the customized integrated catheter can provide similar electrical recordings as a standard catheter.

1. **PSOCT measured wall thickness at lesion locations prior to RFA**

Cardiac wall thickness at each ablation location was also evaluated before the delivery of RF energy. All thicknesses are shown on a map in supplementary fig. S6. It was observed that PSOCT could image transmurally at a majority of the locations (31/38, 82%). The LA wall was thicker at the roof and around the mitral annulus. Furthermore, the wall thickness varied at a similar location in different hearts. In some individuals, the thickness at the junction of the left PV and posterior wall and inferior middle posterior wall was beyond the imaging depth range.

**
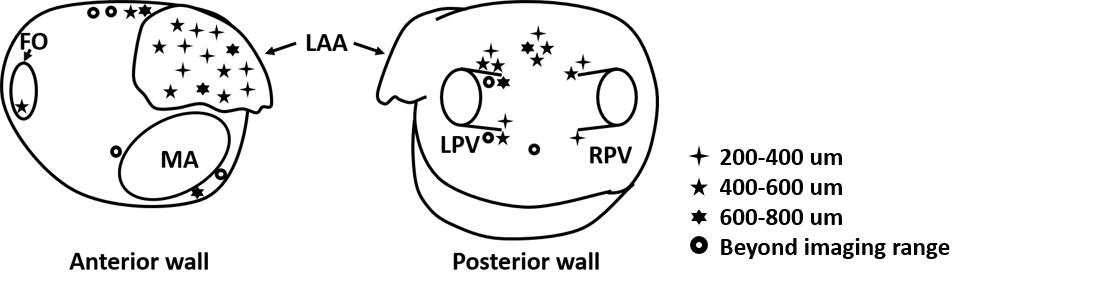
**

**Supplementary figure S6. PSOCT measured wall thickness at lesion locations before RFA. LAA, left atrial appendage; LPV, left PV; RPV, right PV; MA, mitral annulus; FO, foraman ovale.**

1. **PSOCT lesion quality monitoring validation map**

The transmurality of the 24 lesions with analyzable datasets was analyzed for lesion transmurality with PSOCT images and TTC staining. A map of the results is shown in supplementary fig. S7. Nineteen of the lesions were identified as transmural by TTC staining, of which 17 lesions were also identified as transmural with PSOCT monitoring. The other 2 were mis-classified as nontransmural lesions with PSOCT monitoring, both of which were located at superior LAA. 5 lesions were identified as non-transmural with both TTC staining and PSOCT monitoring.

**
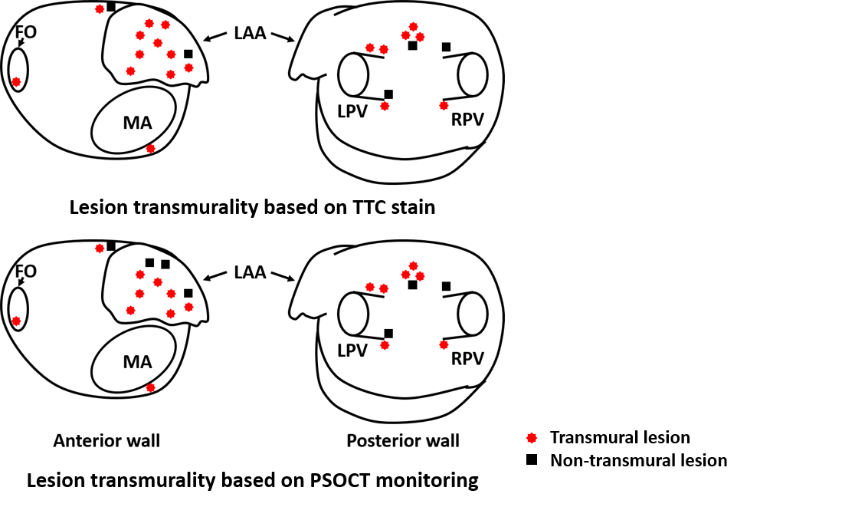
**

**Supplementary figure S7. Lesion quality monitor validation map**

**Reference:**

1. Godfraind-De Becker A, Gillis JM: Analysis of the birefringence of the smooth muscle anococcygeus of the rat, at rest and in contraction. I. J Muscle Res Cell Motil 1988; 9:9–17.
